# Knowledge, attitudes, self-reported competencies and perceived barriers regarding evidence-based practice among undergraduate midwifery students in Germany: a cross-sectional study

**DOI:** 10.1186/s12909-026-10846-y

**Published:** 2026-11-13

**Authors:** Grace Komuhangi, Florian Neuhann, Valerie R. Louis, Brian Mikka, Anne C. Manawa Nougho, Jürgen Wacker

**Affiliations:** 1 Faculty of Medicine and University Hospital, Heidelberg Institute of Global Health (HIGH), Heidelberg University, Heidelberg, Baden-Württemberg Germany; 2 School of Medicine and Health Sciences, Levy Mwanawasa Medical University, Lusaka, Zambia; 3 Department of Clinical Epidemiology, College of Health Sciences, School of Medicine, Makerere University, Kampala, Uganda; 4 Midwifery Research and Education Unit, Department of Obstetrics, Gynecology and Reproductive Medicine, Hannover Medical School (MHH), Hannover, Germany; 5 Department of Obstetrics and Gynecology (Universitäts-Frauenklinik), Heidelberg University Hospital, Heidelberg, Baden-Württemberg Germany

**Keywords:** Evidence-based practice, Midwifery education, Undergraduate students, Knowledge, Attitudes, Barriers

## Abstract

**Background:**

In 2020, Germany transitioned from vocational midwifery training to a dual bachelor’s programme. Evidence-based practice (EBP) was established as a required learning outcome; however, data remain limited regarding students’ EBP knowledge, attitudes, self-reported competencies, and perceived barriers. This study examined these outcomes and their associated factors.

**Methods:**

A cross-sectional online survey was conducted from January to June 2025 among midwifery students at seven universities in five German states. Eligible participants were in semester 2 or later and had completed an EBP or research methods module as well as a clinical placement. The German-language questionnaire was adapted from the Evidence-Based Practice Questionnaire and its student version. The primary outcome was recognition of the comprehensive definition of EBP from a single multiple-choice item (good EBP knowledge). Secondary outcomes included self-reported EBP competence, attitudes, and perceived barriers. Prevalence ratios with 95% confidence intervals (CI) were estimated using modified Poisson regression.

**Results:**

Among 272 students, 64.9% (95% CI 59.1 to 70.4) demonstrated good EBP knowledge, 54.8% (48.8 to 60.6) reported ability in at least three of four EBP activities, 59.5% (53.5 to 65.2) exhibited a positive attitude, and 65.8% (59.9 to 71.2) reported high perceived barriers. While attitudes were generally favourable, EBP behaviours were reported less frequently. The primary barriers identified were limited proficiency in scientific English, insufficient support at placement sites, and difficulty translating evidence into practice, with ratings concentrated at the upper end of the scale. Semester of study was most strongly associated with good EBP knowledge, whereas associations with learning and placement settings diminished after adjustment. The semester gradient remained after adjusting for university and after excluding the 36.4% of participants with uniform barrier ratings, which reduced all prevalence estimates by 13 to 20% points.

**Conclusions:**

Approximately two-thirds of students recognised the comprehensive definition of EBP, and just over half reported ability in most assessed EBP activities and a positive attitude. Recognition was more prevalent in later semesters; however, the cross-sectional design does not allow for separation of educational progression from cohort or institutional differences. Competence was self-reported, and prevalence estimates were sensitive to uniform responding. These findings from a self-selected sample provide baseline data for a midwifery education system undergoing transition.

**Supplementary Information:**

The online version contains supplementary material available at https://doi.org/10.1186/s12909-026-10846-y.

## Background

Evidence-based practice (EBP) integrates the best available research evidence with clinical expertise and patient preferences to optimise clinical outcomes [1, 2]. Recent literature increasingly emphasises shared decision-making and the contextual knowledge contributed by both practitioners and women, positioning EBP as a deliberative process with the woman rather than a simple application of research findings [3, 4]. In midwifery, EBP is fundamental for ensuring safe, effective, and individualised care for childbearing women and their families [5]. Globally, EBP is recognised as a core competency for healthcare professionals, with growing emphasis on embedding EBP principles into undergraduate curricula [6]. The need for EBP is particularly pronounced in midwifery, where care during pregnancy, labour, and the postnatal period exhibits substantial and persistent variation across settings and includes routine interventions whose benefit for healthy women is not established. The ability to locate, appraise, and apply evidence directly determines whether care supports physiological processes or introduces interventions without clear indication. Midwives operate at the intersection of independent practice and interprofessional decision-making, making the capacity to justify actions based on evidence, rather than local custom, central to professional accountability and women’s informed choice.

In 2020, Germany enacted a comprehensive reform of midwifery education. The Midwifery Act (Hebammengesetz) and the Study and Examination Regulation for Midwives (Studien- und Prüfungsverordnung für Hebammen, HebStPrV) replaced the previous three-year vocational training model with a university-based bachelor’s programme, aligning German midwifery education with EU Directive 2013/55/EU and wider European Union standards [7–11]. The programme adopts a dual structure, lasting six to eight semesters and comprising at least 4,600 h. Of these, at least 2,200 h are dedicated to university-based theoretical and practical instruction, including skills-laboratory teaching, and at least 2,200 h are allocated to supervised clinical placements in hospitals, out-of-hospital midwife-led settings, and self-employed midwifery practice. Students maintain a training contract with a clinical partner throughout the programme, culminating in a state examination that confers both a Bachelor of Science degree and professional licensure. Anlage 1 of the HebStPrV specifies the required competencies, including research literacy and evidence-based care.

The newly established academic midwifery programmes in Germany employ contemporary pedagogical techniques to develop EBP competencies. These methods include problem-based learning, experiential learning, and blended learning approaches, which are intended to promote analytical thinking, scientific reasoning, and the integration of theory and practice [12]. However, the effectiveness of these educational strategies and the extent of EBP competencies attained by students remain largely unexamined in the German context.

Previous research in nursing and other healthcare professions indicates that, although students often develop positive attitudes towards EBP during their education, substantial gaps frequently persist between knowledge and practice [13]. Barriers such as time constraints, limited access to resources, insufficient critical appraisal skills, and lack of organisational support have been identified as significant obstacles to EBP implementation [14, 15].

Evidence specific to midwifery education remains limited. A mixed-method systematic review of 59 studies in midwifery and nursing education identified numerous strategies for integrating research, teaching, and EBP, but found no conclusive evidence regarding the most effective approach; the divide between theory and practice was a consistent theme [3]. Reviews of studies involving pre-registration midwifery and nursing students indicate that instruction on research and EBP improves students’ knowledge and attitudes [16], yet students may value this instruction less than clinical content and often perceive a disconnect between university teaching and clinical practice [17]. Qualitative studies have examined how midwifery students experience EBP in both academic and clinical settings [18], and frameworks for research-inspired teaching have been proposed to integrate research and EBP throughout midwifery and nursing curricula [19, 20]. Quantitative data on EBP knowledge, attitudes, and barriers among undergraduate midwifery students are scarce, and none are available for students enrolled in the new academic programmes in Germany.

Students, rather than qualified midwives, constitute the appropriate population for this investigation for three reasons. First, students are directly exposed to the reformed curriculum, whereas most practising midwives in Germany were trained under the previous vocational system, resulting in different competencies. Second, students possess clinical experience; under the dual structure, they practise under supervision throughout the programme, and the state examination assesses EBP competencies. Third, pre-registration education is the stage at which EBP habits are established, and curricula can still be adjusted, making baseline data on students essential for curriculum committees. A follow-up study among qualified midwives is planned to assess graduates’ EBP competencies in professional practice.

This study therefore aimed (i) to determine the prevalence of recognition of the comprehensive definition of EBP, referred to as good EBP knowledge, and the factors associated with it (main objective), and (ii) to describe attitudes towards EBP, self-reported ability in EBP activities, EBP-related behaviours, and perceived barriers to EBP, as well as to examine factors associated with self-reported ability and a positive attitude (secondary objectives) among undergraduate midwifery students in Germany.

## Methods

### Study design and setting

This cross-sectional survey formed part of a mixed-methods doctoral project examining modern teaching strategies in undergraduate midwifery education and their relationship with evidence-based practice (EBP) competencies [21]. The study was conducted from January to June 2025 at seven German universities with accredited undergraduate midwifery programmes in Bavaria, Rhineland-Palatinate, Berlin, Lower Saxony, and North Rhine-Westphalia. Reporting follows the Strengthening the Reporting of Observational Studies in Epidemiology (STROBE) guidance for cross-sectional studies [22].

### Educational context

Undergraduate midwifery education in Germany is regulated by the Midwifery Reform Act (Hebammenreformgesetz) and the associated Study and Examination Ordinance for Midwives (Studien- und Prüfungsverordnung für Hebammen, HebStPrV) [11]. The reform established university-based midwifery education leading to a bachelor’s degree and professional qualification, while retaining supervised clinical education as an integral component of the programme.

All participating programmes follow the same national regulations; however, universities differ in how they organise and schedule academic and clinical learning. For example, programmes vary in the timing of research methods and evidence-based midwifery instruction, as well as in how clinical placements are arranged. Previous research has demonstrated that academic midwifery education in Germany has been implemented heterogeneously across institutions since the reform [8]. These institutional differences are important for interpreting associations between academic progression, learning environment, and EBP-related outcomes in this study.

The participating programmes were established during the initial implementation phase of academic midwifery education in Germany, primarily between the winter semesters of 2019/20 and 2020/21. Consequently, the study was conducted while these programmes were still evolving and integrating research methods, EBP, and clinical learning more closely into their curricula.

### Study population, eligibility and recruitment

The study population comprised students enrolled in primary-qualifying B.Sc. midwifery programmes at German universities. Students were eligible if they were at least 18 years old, in their second semester or higher, had completed at least one module on evidence-based practice or research methods, and had undertaken at least one supervised clinical placement. First-semester students were not eligible because they had not yet had sufficient academic and clinical exposure to answer the EBP items. Because these criteria excluded students with the least exposure to EBP teaching, they may have inflated the prevalence estimates; we discuss this in the Limitations. We recorded the semester of study as the semester in which the student was enrolled at the time of the survey and analysed it as a categorical variable. Programmes last 6 to 8 semesters, so semester 8 exists only in 8-semester programmes or for students who extend their studies, which explains why only 1 participant was in semester 8.

We constructed the sampling frame by identifying 48 German universities offering an accredited primary-qualifying B.Sc. midwifery programme, based on the register of the German Midwives Association [23] and published programme descriptions [24]. Seven universities participated in the study.

We applied a proportionate stratified approach at the university level, setting each university’s target number of participants in proportion to its programme enrolment to ensure the sample reflected the distribution of students across institutions. Students within each university were not randomly selected. Recruitment used an anonymous online survey, and participation was voluntary. Thus, the design involved proportionate stratification by institution, followed by voluntary self-selection within strata.

**Fig. 1:**
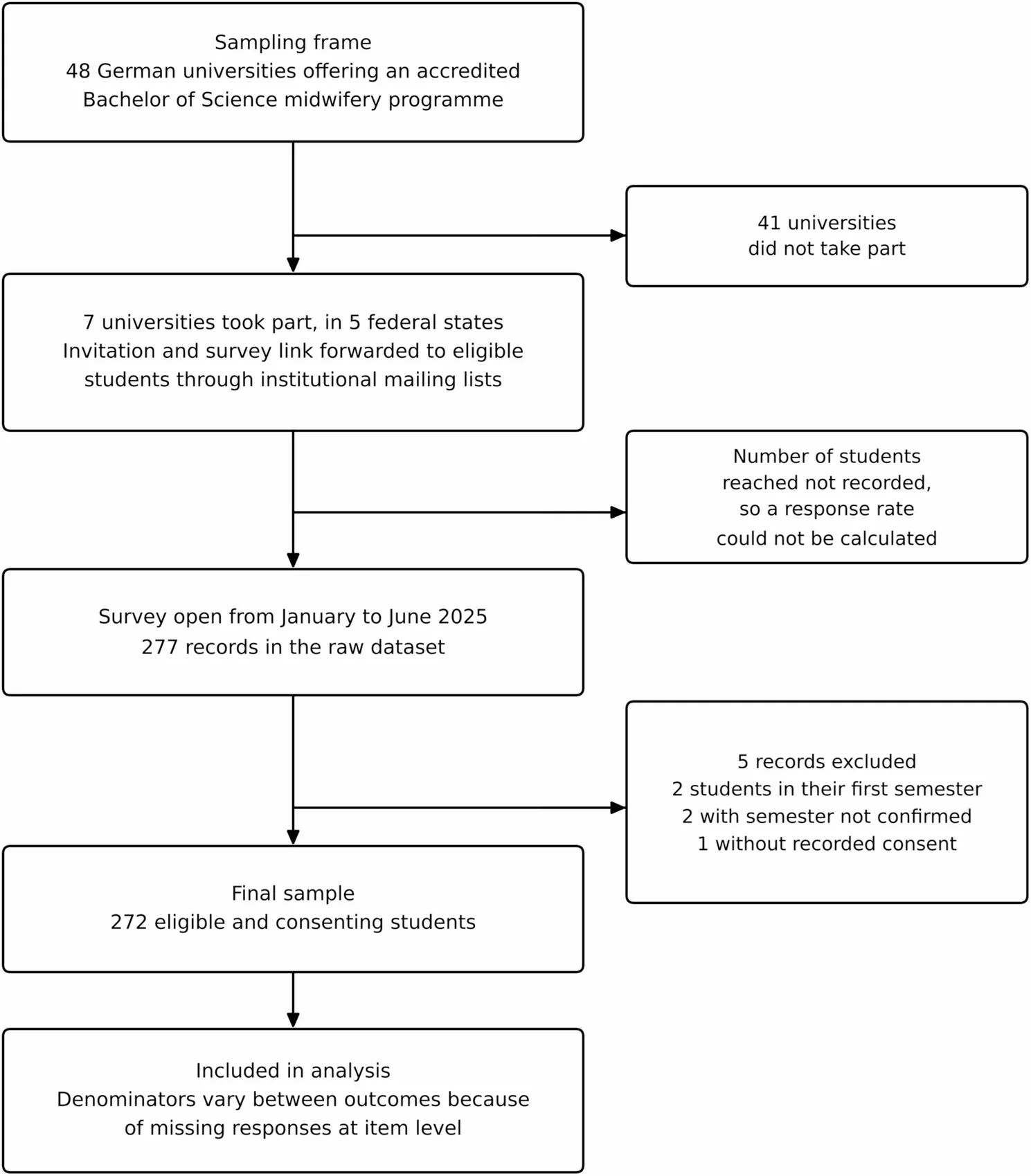
Flow diagram of participant recruitment and inclusion. Students at seven participating universities were invited through programme administrators. Of 277 records in the raw dataset, five were excluded after eligibility and consent had been checked, giving a final sample of 272 students

Recruitment was mediated by participating universities. Programme administrators received a standard invitation from the research team and forwarded it to eligible students via institutional student mailing lists. The invitation described the study and included a link to the online questionnaire. Administrators did not select participants, did not know who responded, and had no access to individual responses or study data. Students were informed that participation was voluntary and anonymous, that declining participation had no consequences, and that responses were not linked to academic assessment. The team offered no financial or other incentives, and students were not recruited directly in classrooms or in person. Because invitations were distributed through institutional mailing lists, the research team could not access mailing records or determine how many eligible students received or opened the invitation; therefore, the team could not calculate a conventional response rate. Fig. 1 summarises the recruitment process, including the number of participating universities and the final sample.

The raw survey dataset contained 277 records. We removed five records after checking eligibility and consent: two from first-semester students, two with an unconfirmed semester, and one without recorded consent. One of the two records with an unconfirmed semester duplicated the answers of three other records from the same university on all but two items but reported a different semester, making it impossible to establish the semester of study. This resulted in a final sample of 272 eligible and consenting students. Denominators differ slightly between outcomes because of item-level missing responses, and we report the denominator alongside each result.

### Sample size

We calculated the required sample size in advance using the Kish–Leslie formula for estimating a population proportion [25]. The calculation used a 95% confidence level (Z = 1.96), an expected proportion of 0.50 and a precision of ± 6% points. We chose 0.50 as a conservative estimate because it yields the largest sample size. These assumptions gave an initial required sample size of 267.

We estimated the population of undergraduate students enrolled in B.Sc. midwifery programmes in Germany during the survey period at approximately 1,280, based on published programme and cohort information [23, 24]. Because this population was finite relative to the initially calculated sample, we applied a finite population correction, resulting in a minimum required sample of 221 participants. To account for incomplete questionnaires and other unusable responses, recruitment aimed to obtain approximately 270 participants.

The final sample of 272 students from seven universities exceeded both the minimum required sample of 221 and the recruitment target of approximately 270. Against an estimated national population of 1,280 students, the sample corresponds to about 21% of that population, and the 95% confidence interval around the primary outcome had a half-width of approximately 5.7% points.

### Data collection instrument

We collected data using a modified version of the Evidence-Based Practice Questionnaire (EBPQ), originally developed and validated by Upton and Upton for nurses and other health professionals [26]. To make it more relevant for undergraduate midwifery students, we also drew on the Student Evidence-Based Practice Questionnaire (S-EBPQ) [27]. The original EBPQ contains 24 items covering practice, attitudes and knowledge or skills. The S-EBPQ, developed for students, informed the wording and structure of several items.

The questionnaire was administered in German. Two bilingual team members (GK and FN) carried out a forward-backwards translation process. Each translated the original instruments into German and then back into English independently. The team discussed differences until they agreed the meaning matched the original, ensuring the translation was accurate and appropriate for German undergraduate midwifery students. This procedure was a forward-and-backwards translation within the research team. It did not constitute a formal cross-cultural adaptation and validation, which would also involve independent translators, an expert committee review of all versions, and psychometric testing of the translated instrument [28].

We made three adaptations for German undergraduate midwifery students. First, we reworded practice-related items to reflect students’ perspectives rather than those of practising clinicians. Second, we removed three items on prescribing and independent clinical decision-making, since these do not form part of undergraduate midwifery education in Germany. Third, we added four items on barriers to evidence-based practice, based on findings from the earlier qualitative phase, to reflect challenges specific to German university-based midwifery education. We otherwise kept item wording as close as possible to the source instruments.

Four experienced midwifery lecturers reviewed the questionnaire for relevance, clarity, completeness and contextual fit. The research team discussed their feedback and made minor wording changes to improve clarity and relevance. A formal Content Validity Index (CVI) was not calculated. The revised questionnaire was pilot tested on 13 January 2025 with 13 undergraduate midwifery students to assess clarity, comprehension, completion time and performance within the REDCap platform. Pilot feedback led to minor wording changes, while the constructs and scoring remained unchanged. Pilot participants were not included in the main survey.

The final questionnaire, as revised after the pilot and administered from January 2025, comprised 41 items organised into five sections: (1) sociodemographic and educational characteristics (7 items); (2) knowledge of EBP (4 items); (3) self-reported EBP-related competencies and behaviours (10 items); (4) attitudes towards EBP (9 items); and (5) perceived barriers to EBP (11 items). In the final section, participants first indicated whether they experienced barriers to applying EBP in practice and then rated the relevance of ten listed barriers on a scale from 1 (not relevant) to 10 (highly relevant). Completion took approximately 15 min.

### Variables and operationalisation

The primary outcome was recognition of the comprehensive definition of EBP, referred to in this paper as good EBP knowledge. We defined good EBP knowledge a priori as correct identification, from a single multiple-choice question, of the internationally accepted definition of EBP as the integration of research evidence, clinical expertise, and patient preferences [1, 2]. We selected this item as the primary outcome for three reasons. First, understanding that EBP integrates all three components is a precondition for subsequent steps in the EBP process, and the attitude results indicate that this integrated understanding cannot be assumed. Second, because the programmes had been running for only a few years and introduced research methods at different points in the curriculum [8], we preferred an item that all eligible students could reasonably answer over performance tests that presuppose completed teaching in searching and appraisal. Third, a binary outcome enabled comparison with earlier studies using the EBPQ. Performance-based instruments such as the Fresno test [29] and the Berlin questionnaire [30] assess the EBP process more comprehensively, but the Fresno test requires open answers scored by trained raters; both instruments were developed for medical trainees and assume familiarity with statistical concepts beyond students in the early semesters, and either would have substantially lengthened the questionnaire. The item measures recognition of a definition, not the ability to formulate questions, search for and appraise evidence, or apply it in practice. The paper uses the term knowledge in this narrower sense throughout.

The knowledge section also included self-assessment items regarding participants’ understanding of the EBP model and their ability to describe the steps of the EBP process. The competence section asked participants to rate their ability to identify the most appropriate study design to evaluate calcium supplementation for preventing pre-eclampsia. These items assessed students’ confidence rather than their actual performance. Consequently, the definition item was the only objective knowledge measure, so we could not construct a composite objective knowledge score. To provide a broader perspective, the Results report the two self-assessment items on EBP knowledge, and Table 5 presents the study design item.

We measured self-reported EBP-related competencies, behaviours, and attitudes using Likert-scale items from the modified EBPQ. Item scores were summed to generate overall scores for each domain. The attitude scale comprised nine items scored from 1 (least favourable) to 5 (most favourable), resulting in a possible range of 9 to 45. The self-reported competence domain included four practical ability items scored on the same five-point scale, yielding a possible range of 4 to 20. These four items were selected from the knowledge and skills domain of the EBPQ because they describe the practical steps of searching, appraisal, and study design taught in the programmes. Because all competence and attitude items are self-report, they measure students’ confidence and perspectives rather than objectively assessed competence. The Data Analysis section reports internal consistency and the cut-off values used to classify participants, and Tables 4 and 5 present item-level distributions.

The explanatory variables included age, gender, university, country where the pre-university qualification was obtained, semester of study, main clinical placement setting, and whether placements had been completed in four clinical environments: university hospitals, private hospitals, midwife-led birth centres, and self-employed midwifery practice.

We measured clinical placement experience with a question allowing participants to select all settings in which they had completed placements. Each setting was coded as a separate binary variable; therefore, percentages for placement settings do not sum to 100.

### Data collection procedure

We collected data using an online survey administered through REDCap [31, 32], a platform compliant with the General Data Protection Regulation (GDPR) [33] and hosted on institutional servers with encrypted data transmission. The survey remained open from January to June 2025. After data collection, we reviewed the dataset during cleaning to identify potential duplicate submissions. Because the survey was anonymous, we could not attribute records with identical answers to every item with certainty to repeated submissions; we retained these records in the main analysis and examined their influence in a sensitivity analysis.

### Ethical considerations

The study received ethical approval from the Ethics Committee of the Medical Faculty, University of Heidelberg (approval number S-709/2024, dated 23 December 2024). All procedures adhered to the Declaration of Helsinki (2013 revision) [34] and to the German Federal Data Protection Act [35]. We obtained informed consent electronically from all participants before data collection.

### Data analysis

We analysed data from 272 eligible participants using Stata version 15 (StataCorp, College Station, Texas, United States). The analysis followed the research objectives. We primarily examined the prevalence of good EBP knowledge and the factors associated with it. We examined attitudes towards EBP, self-reported EBP-related competencies and behaviours, and perceived barriers as secondary outcomes. Denominators are reported wherever they differ from 272.

We defined good EBP knowledge before analysis as correctly selecting the comprehensive definition of EBP from a multiple-choice question. This definition included scientific literature, specialist staff practical experience, and client preferences as the basis for daily practice. Partial definitions did not meet the criterion. We summarised participant characteristics as means and standard deviations for continuous variables and frequencies and proportions for categorical variables. We calculated confidence intervals for proportions using the Wilson method [36].

We used modified Poisson regression with robust variance [37, 38] to estimate associations with the primary outcome and reported crude and adjusted prevalence ratios (cPR and aPR) with 95% confidence intervals. We chose prevalence ratios over odds ratios because the outcome was common and odds ratios can overstate associations in this context. Candidate variables included age, semester of study, main area of midwifery work, and clinical placement experience in four settings. We retained all candidate variables in the multivariable model to ensure each had an adjusted estimate, and reported all results regardless of statistical significance, in accordance with item 16 of the STROBE statement. Semester five, the largest group, served as the reference category. University was not included in the main model, as it defined the sampling strata, was not recorded for 15 participants, and three recorded institutions contributed fewer than 10 participants each, which would have resulted in sparse cells and imprecise estimates. University was also associated with semester (Cramér’s V 0.23, *p* < 0.001): for example, 23 of the 25 participants in semester 2 came from two universities, and one university contributed no participants in semesters 2 or 7. Because universities differ in curriculum design, timing of EBP teaching, placement models, and support structures [8], omitting university may leave residual confounding. Therefore, we conducted a sensitivity analysis in which we added university as a fixed effect, combining the three smallest recorded institutions among participants with university recorded. With only seven sites, we did not use cluster-robust standard errors because they are unreliable with few clusters. We considered gender but did not model it, since 263 of 271 participants were female; similarly, we did not model country of pre-university qualification, as only two participants qualified outside Germany and both met the outcome, producing complete separation. We included the single participant in semester eight in the descriptive analysis but not in the regression models, since the semester grouping ends at semester seven. Two further sensitivity analyses repeated the prevalence estimates and the primary model after excluding, first, participants who gave the same rating to all 10 barrier items (uniform responders) and, second, records that were exact copies of an earlier record on every item. A post hoc sensitivity analysis of the threshold for the competence outcome is described below. We conducted the sensitivity analyses with the same model specification using the statsmodels package in Python 3.

Three secondary outcomes were derived. We based self-reported ability in EBP activities on four practical ability items: applying search strategies, critically appraising the literature, identifying the appropriate study design, and critically evaluating a research article. Each item was counted as endorsed when participants answered “agree” or “strongly agree” (for the item on critically evaluating a research article, “applies” or “fully applies”). As in the original analysis plan, participants endorsing at least three of the four items met the study-defined criterion, referred to as self-reported ability in at least three of four EBP activities. This threshold represented endorsement of the majority of the assessed abilities; investigators set it, and it is not a validated competence cut-off, and Table 5 shows results by item. Missing responses were never counted as agreement, and participants were classified only when the available responses determined the result, so all 272 participants could be classified. A post hoc sensitivity analysis applied the stricter requirement of endorsement of all four items and repeated the prevalence estimate and the regression model; under this criterion, two participants with one missing item could not be classified. We measured attitudes towards EBP by summing the nine attitude items, each scored from 1 (least favourable) to 5 (most favourable), for a total score between 9 and 45. We classified a participant as having a positive attitude if the mean item score was 4 or higher, that is, if the average response was at least “agree”. We derived this threshold from the response scale and set it for the present analysis rather than pre-specifying it in the protocol. We assessed internal consistency with Cronbach’s alpha for all composite scales: 0.97 for the nine attitude items, 0.96 for the four practical ability items, and 0.95 for the eight substantive barrier items. Values this high suggest some redundancy between items and may also reflect uniform responding, rather than high reliability alone [39]. Beyond internal consistency, we conducted no further psychometric evaluation, such as factor analysis or test-retest reliability. We defined high perceived barriers as a rating of 8 to 10 on the 1 to 10 relevance scale for at least six of the eight substantive barrier items, excluding items on the feasibility of introducing EBP and lack of interest, since these do not describe external obstacles. We estimated associations with self-reported ability in at least three of four EBP activities and with a positive attitude using modified Poisson regression, as in the primary model. We described perceived barriers at the item level and did not model them. Because barrier ratings were concentrated at the upper end of the scale, we report item means and standard deviations alongside medians, and we counted the number of participants who gave the same rating to all items to assess uniform responding.

A three-level version of the competence score was considered but is not reported here. The distribution was strongly bimodal, and the binary models used to check the proportional odds assumption did not converge because of separation, so we could not test the assumption reliably. The binary composite defined above is reported instead.

We examined missing data for every variable before analysis; missingness was low, with at most 15 of 272 missing for any variable and at most 7 of 272 missing for any outcome item. No imputation was performed. Each analysis used complete cases for the variables involved, and we did not recode missing responses as unfavourable. To examine whether missingness related to stage of study, we compared the proportion of participants with at least one missing value among the outcome and model variables across semesters. This proportion ranged from 0% in semester 3 to 8.2% in semester 4 and did not differ by semester (chi-square test with permutation p-value, *p* = 0.61). Given the low level of missingness, we did not perform multiple imputation. All tests were two-sided with a significance level of 0.05.

## Results

### Characteristics of study participants

Table 1 presents the characteristics of the 272 study participants. The mean age was 23.0 years (SD 2.2), and 263 of 271 participants (97.0%) were female. Participants were enrolled in semesters two through eight, with the largest proportions in semester five (*n* = 64, 23.5%) and semester six (*n* = 55, 20.2%). Two institutions accounted for 140 participants (54.5% of the 257 for whom institutional data were available). Nearly all participants obtained their pre-university qualification in Germany (*n* = 260 of 262, 99.2%). Clinical placement experience was extensive: 257 participants (94.5%) completed a placement in a private hospital, 246 (90.4%) in a university hospital, 143 (52.6%) in self-employed midwifery practice, and 127 (46.7%) in a midwife-led birth centre.

**Table 1:** Characteristics of the study participants (*N* = 272)

Characteristic	*n* (%)	Valid *n*
Age, years, mean (SD)	23.0 (2.2)	272
Gender		271
Female	263 (97.0)	
Male	5 (1.8)	
Diverse	3 (1.1)	
Institution		257
Public university of applied sciences, Rhineland-Palatinate	84 (32.7)	
Private university of applied sciences, Bavaria	56 (21.8)	
Public university, Lower Saxony (1)	36 (14.0)	
Public university of applied sciences, Lower Saxony (2)	36 (14.0)	
Public university of applied sciences, Bavaria	32 (12.5)	
Private university of applied sciences, North Rhine-Westphalia	8 (3.1)	
Public university, North Rhine-Westphalia b	3 (1.2)	
Public university, Berlin	2 (0.8)	
Pre-university qualification obtained		262
Germany	260 (99.2)	
Outside Germany	2 (0.8)	
Semester of study		272
Semester 2	25 (9.2)	
Semester 3	34 (12.5)	
Semester 4	49 (18.0)	
Semester 5	64 (23.5)	
Semester 6	55 (20.2)	
Semester 7	44 (16.2)	
Semester 8	1 (0.4)	
Area in which most work was carried out		268
Mother and child health care	146 (54.5)	
Theoretical and practical teaching events	122 (45.5)	
Clinical placement completed a		272
Private hospital	257 (94.5)	
University hospital	246 (90.4)	
Self-employed midwifery practice	143 (52.6)	
Midwife-led birth centre	127 (46.7)	

Age is reported as mean (SD); all other rows as n (%). Percentages are calculated on the valid n for each characteristic. a Participants could select more than one setting, so these percentages do not sum to 100. b Three respondents recorded an institution that was not among the seven participating study sites; these responses were retained in the analysis sample

*SD *standard deviation

### Prevalence of the study outcomes

Table 2 summarizes the prevalence of primary and secondary outcomes. Among 271 participants who provided a valid response to the EBP definition item, 176 (64.9%, 95% CI 59.1 to 70.4) selected the comprehensive definition and were classified as having good EBP knowledge. Self-reported ability in at least three of four EBP activities was observed in 149 of 272 participants (54.8%, 95% CI 48.8 to 60.6), while a positive attitude towards EBP was reported by 160 of 269 participants (59.5%, 95% CI 53.5 to 65.2). High perceived barriers were noted in 177 of 269 participants (65.8%, 95% CI 59.9 to 71.2).

**Table 2:** Prevalence of the primary and secondary outcomes

Outcome	Classifiable, *n*	Present, *n*	% (95% CI)
Good EBP knowledge (primary) a	271	176	64.9 (59.1 to 70.4)
Self-reported ability in at least three of four EBP activities b	272	149	54.8 (48.8 to 60.6)
Positive attitude towards EBP c	269	160	59.5 (53.5 to 65.2)
High perceived barriers d	269	177	65.8 (59.9 to 71.2)

Confidence intervals were calculated by the Wilson method. a Correct identification of the comprehensive definition of EBP. b Endorsement of at least three of four practical ability items; the threshold was set by the investigators and is not a validated competence cut-off. c Mean score of at least 4 across the nine attitude items. d At least six of eight substantive barriers rated 8 to 10. Three participants could not be classified

*CI *confidence interval

Self-assessed knowledge demonstrated a similar distribution. In total, 133 of 272 participants (48.9%) reported feeling sure or very sure of their understanding of the EBP model, and 125 (46.0%) expressed confidence in their ability to describe the steps of the EBP process. Among students in semesters 2 to 4, 4 of 108 (3.7%) and 3 of 108 (2.8%) reported such confidence, compared with 129 of 163 (79.1%) and 122 of 163 (74.8%) in semesters 5 to 7.

### Factors associated with good EBP knowledge

Table 3 presents the crude and adjusted prevalence ratios for good EBP knowledge. The prevalence differed markedly between semesters, from 1 of 25 participants (4.0%) in semester two and 4 of 34 (11.8%) in semester three, to 26 of 49 (53.1%) in semester four, 50 of 64 (78.1%) in semester five, 53 of 55 (96.4%) in semester six, and 42 of 44 (95.5%) in semester seven. In the multivariable analysis, semester of study was the only variable that remained significantly associated with good EBP knowledge. Relative to semester five, prevalence was lower in semester two (aPR 0.05, 95% CI 0.01 to 0.37), semester three (aPR 0.17, 95% CI 0.06 to 0.44), and semester four (aPR 0.70, 95% CI 0.49 to 0.99), and higher in semester six (aPR 1.21, 95% CI 1.01 to 1.46). The estimate for semester seven was comparable but less precise (aPR 1.23, 95% CI 0.97 to 1.56). Crude associations with area of work, placement experience in self-employed midwifery practice, and midwife-led birth centre were not sustained after adjustment. Likewise, the crude association with age (1.11 per year, 95% CI 1.07 to 1.16) was attenuated after adjustment (1.01, 95% CI 0.95 to 1.08).

**Table 3:** Factors associated with good EBP knowledge (*n* = 267 in the multivariable model)

Variable	cPR (95% CI)	*p*	aPR (95% CI)	*p*
Age, per year	1.11 (1.07 to 1.16)	< 0.001	1.01 (0.95 to 1.08)	0.695
Semester of study				
Semester 5 (reference)	1.00		1.00	
Semester 2	0.05 (0.01 to 0.35)	0.002	0.05 (0.01 to 0.37)	0.003
Semester 3	0.16 (0.06 to 0.39)	< 0.001	0.17 (0.06 to 0.44)	< 0.001
Semester 4	0.68 (0.51 to 0.91)	0.010	0.70 (0.49 to 0.99)	0.045
Semester 6	1.23 (1.07 to 1.42)	0.003	1.21 (1.01 to 1.46)	0.037
Semester 7	1.22 (1.06 to 1.41)	0.007	1.23 (0.97 to 1.56)	0.093
Area in which most work was carried out				
Mother and child health care (reference)	1.00		1.00	
Theoretical and practical teaching events	0.42 (0.33 to 0.53)	< 0.001	0.72 (0.50 to 1.04)	0.079
Placement in self-employed midwifery practice				
Yes (reference)	1.00		1.00	
No	0.47 (0.38 to 0.58)	< 0.001	1.23 (0.87 to 1.72)	0.236
Placement in a university hospital				
Yes (reference)	1.00		1.00	
No	0.85 (0.59 to 1.22)	0.376	0.84 (0.56 to 1.24)	0.380
Placement in a private hospital				
Yes (reference)	1.00		1.00	
No	0.87 (0.55 to 1.39)	0.569	1.00 (0.66 to 1.53)	0.982
Placement in a midwife-led birth centre				
Yes (reference)	1.00		1.00	
No	0.50 (0.42 to 0.61)	< 0.001	1.12 (0.87 to 1.45)	0.368

Estimates were obtained using modified Poisson regression with robust variance. Statistical significance was defined as *p* < 0.05. All candidate variables were retained in the multivariable model to ensure each variable received an adjusted estimate. Estimates for semesters two and three are based on small numbers of participants with the outcome, resulting in reduced precision

*cPR* crude prevalence ratio, *aPR* adjusted prevalence ratio, *CI* confidence interval

Sensitivity analysis including university as a covariate demonstrated that the adjusted prevalence ratios for semester were consistent with those observed in the primary model: 0.06 (95% CI 0.01 to 0.42) for semester two, 0.21 (0.09 to 0.49) for semester three, 0.75 (0.53 to 1.06) for semester four, 1.16 (0.97 to 1.39) for semester six, and 1.21 (0.94 to 1.55) for semester seven. No other estimates changed materially.

A total of 99 participants (36.4%) provided identical ratings for all 10 barrier items, with 97 assigning the maximum score of 10. These uniform responders were primarily in semesters four to seven and were more likely than other participants to meet each outcome definition. Excluding these individuals reduced the prevalence of good EBP knowledge to 52.0% (95% CI 44.6 to 59.3), self-reported ability in at least three of four EBP activities to 42.2% (35.1 to 49.6), positive attitude to 45.3% (38.1 to 52.8), and high perceived barriers to 46.2% (39.0 to 53.7). The semester gradient remained evident (semester two aPR 0.06, 95% CI 0.01 to 0.43; semester three aPR 0.18, 0.07 to 0.48; semester six aPR 1.27, 1.00 to 1.60). The dataset also included 16 groups of 2 to 4 records (43 records in total) with identical responses to every item, all from uniform responders. One additional record, which duplicated the answers of one group but reported a different semester, had already been excluded during data cleaning. Excluding the 27 subsequent duplicates resulted in a prevalence of good EBP knowledge of 61.2% (95% CI 55.0 to 67.1) and semester estimates similar to those in the main model.

### Factors associated with the secondary outcomes

After adjustment, age remained significantly associated with both outcomes (competence aPR 1.12 per year, 95% CI 1.06 to 1.19; attitude aPR 1.08, 95% CI 1.03 to 1.14). Participants who spent most of their time in theoretical and practical teaching events were less likely than those working mainly in mother and child health care to meet the competence criterion (aPR 0.41, 95% CI 0.26 to 0.66) or to have a positive attitude (aPR 0.62, 95% CI 0.39 to 0.99). Similarly, participants without placement experience in self-employed midwifery practice were less likely to meet the competence criterion (aPR 0.39, 95% CI 0.22 to 0.71) or to have a positive attitude (aPR 0.59, 95% CI 0.37 to 0.94). Placement outside a university hospital was associated with a higher prevalence of the competence outcome (aPR 1.95, 95% CI 1.39 to 2.74); however, only 26 participants had not been placed in a university hospital. The semester gradient observed for knowledge was less consistent for the secondary outcomes. After adjustment, only semester four differed from semester five for competence (aPR 0.27, 95% CI 0.13 to 0.55), and only semester three for attitude (aPR 0.38, 95% CI 0.14 to 0.99). No participant in semester two met the attitude outcome; therefore, this estimate was not calculated. Whether the associations with the competence outcome persisted under a stricter definition is reported below (Table 7).

### Attitudes toward evidence-based practice

Table 4 presents attitudes toward evidence-based practice (EBP), which were favorable across all nine items. The highest levels of agreement were observed for statements regarding the need to strengthen personal use of EBP (*n* = 258, 94.9%), the belief that EBP improves patient outcomes (*n* = 253, 93.0%), and the perception that literature and research are useful in daily midwifery practice (*n* = 245, 90.1%). The lowest agreement and most frequent neutral responses were recorded for statements describing the EBP model’s content: specifically, that EBP considers patient preferences and values (*n* = 185, 68.0%, with 81 neutral responses) and that it incorporates clinical experience (*n* = 190, 70.4%, with 76 neutral responses). Explicit disagreement was rare, with no more than 6 participants (2.2%) disagreeing with any item.

**Table 4:** Attitudes towards EBP

Attitude item	Valid *n*	Agree, *n* (%)	Neutral, *n* (%)	Disagree, *n* (%)
I need to strengthen my use of EBP in daily practice	272	258 (94.9)	9 (3.3)	5 (1.8)
EBP improves outcomes for patients	272	253 (93.0)	18 (6.6)	1 (0.4)
Literature and research are useful for EBP in daily practice	272	245 (90.1)	26 (9.6)	1 (0.4)
EBP should be a core component of midwifery studies	272	244 (89.7)	26 (9.6)	2 (0.7)
The use of EBP is necessary in midwifery practice	271	239 (88.2)	31 (11.4)	1 (0.4)
Stronger EBP competence improves my work as a midwife	272	218 (80.1)	52 (19.1)	2 (0.7)
EBP is an important part of midwifery practice	272	206 (75.7)	65 (23.9)	1 (0.4)
EBP takes account of clinical experience	270	190 (70.4)	76 (28.1)	4 (1.5)
EBP takes account of patient preferences and values	272	185 (68.0)	81 (29.8)	6 (2.2)

The ‘agree’ category consolidates the two agreement options, while the ‘disagree’ category consolidates the two disagreement options from the five-point response scale

### Self-reported evidence-based practice competencies and behaviours

Table 5 presents self-reported competencies and behaviours. Approximately half to three-fifths of participants reported each of the four practical abilities, ranging from applying search strategies in scientific databases (*n* = 159, 58.5%) to critically evaluating a research article (*n* = 141, 51.8%). Reported behaviours were consistently less favourable than reported abilities. While 144 participants (52.9%) agreed that research findings should often be used as a basis for practice during placements, only 33 (12.2%) always or frequently critically appraised the literature they used, and only 23 (8.5%) always or frequently applied research findings in practice. In contrast, 119 (44.1%) rarely or never appraised the literature they used.

**Table 5:** Self-reported EBP-related competencies and behaviours

Item	Valid *n*	Favourable, *n* (%)	Neutral, *n* (%)	Unfavourable, *n* (%)
Practical ability (agreement)				
Can apply search strategies in scientific databases	272	159 (58.5)	79 (29.0)	34 (12.5)
Can critically appraise literature for quality and significance	268	148 (55.2)	62 (23.1)	58 (21.6)
Can identify the appropriate study design	272	143 (52.6)	71 (26.1)	58 (21.3)
Can critically evaluate a research article	272	141 (51.8)	49 (18.0)	82 (30.1)
Beliefs and behaviour during clinical placements				
Considers frequent use of research findings important c	272	144 (52.9)	41 (15.1)	87 (32.0)
Critically appraises the literature used a	270	33 (12.2)	118 (43.7)	119 (44.1)
Applies research findings in practice a	272	23 (8.5)	178 (65.4)	71 (26.1)
Confidence and belief				
EBP improves outcomes for mothers and children	270	205 (75.9)	54 (20.0)	11 (4.1)
Confidence in searching for scientific evidence b	270	123 (45.6)	69 (25.6)	78 (28.9)
Confidence in conducting scientific work b	271	132 (48.7)	20 (7.4)	119 (43.9)

a Favourable denotes “always” or “frequently”, neutral “sometimes” and unfavourable “rarely” or “never”. b Favourable denotes the highest confidence category and unfavourable the two lowest. c This item asked whether it is important to use research findings often as a basis for practice during placements and was answered on an agreement scale; it records a belief rather than actual use

### Perceived barriers to evidence-based practice

Table 6 summarizes the perceived barriers. These findings are based on descriptive frequencies rather than regression analysis. The three most frequently cited barriers were limited English-language skills (*n* = 236, 87.4% rated 8 to 10), lack of support from colleagues at the placement site (*n* = 232, 86.2%), and difficulty applying literature findings to practice (*n* = 229, 85.4%). Each of these barriers had a median rating of 10, with mean scores of 9.2, 9.1, and 9.0, respectively. The remaining seven barriers, which included time constraints, insufficient institutional resources, limited competence in searching, appraisal and technology use, lack of interest, and concerns regarding the feasibility of implementing evidence-based practice, were each rated 8 to 10 by 67% to 71% of participants, with mean scores ranging from 8.0 to 8.2. Ratings were concentrated at the upper end of the scale: 132 participants (48.5%) assigned the maximum rating of 10 to at least 8 of the 10 items, and 99 (36.4%) assigned the same rating to all 10 items. Among the remaining respondents, the same three barriers received the highest ratings (8 to 10 by 76.3% to 80.9%), while each of the other items was rated 8 to 10 by approximately half of the participants.

**Table 6:** Perceived barriers to the application of EBP, rated from 1 to 10

Barrier	Valid *n*	Median (IQR)	Mean (SD)	Rated 8 to 10, *n* (%)
Limited English-language skills	270	10 (10 to 10)	9.2 (2.2)	236 (87.4)
No support from colleagues at the placement site	269	10 (10 to 10)	9.1 (2.1)	232 (86.2)
Difficulty transferring literature findings into practice	268	10 (9 to 10)	9.0 (2.0)	229 (85.4)
Insufficient time	268	9 (6 to 10)	8.2 (2.4)	189 (70.5)
Lack of institutional resources (computers, databases)	265	10 (6 to 10)	8.2 (2.5)	185 (69.8)
Limited competence in systematic literature searching	267	10 (6 to 10)	8.1 (2.8)	184 (68.9)
Limited competence in using computers and technology	265	10 (6 to 10)	8.0 (2.8)	181 (68.3)
Limited competence in critically evaluating literature	268	10 (6 to 10)	8.2 (2.6)	182 (67.9)
Introducing EBP is not feasible in midwifery practice	267	9 (6 to 10)	8.1 (2.6)	181 (67.8)
Lack of interest	270	10 (6 to 10)	8.1 (2.8)	182 (67.4)

*IQR* interquartile range, reported as the 25th to the 75th percentile. Higher ratings indicate a barrier perceived as more relevant. SD, standard deviation. Where the median and both quartiles equal 10, ratings were concentrated at the top of the scale, that is, at least three quarters of participants gave the maximum rating of 10. Means are reported to allow items to be ranked despite this clustering

### Sensitivity analysis of the competence threshold

In the post hoc sensitivity analysis requiring endorsement of all four practical ability items, 128 of 270 classifiable participants (47.4%, 95% CI 41.5 to 53.4) met the criterion, compared with 149 of 272 (54.8%) under the main definition. Responses were strongly bimodal: 112 participants endorsed none of the items, and 128 endorsed all four, so 21 participants were reclassified. No participant in semesters two or three, and only one participant in semester four, met the stricter criterion; therefore, semesters two to four were combined in this model. The difference between semesters was preserved and more pronounced (semesters two to four compared with semester five, aPR 0.09, 95% CI 0.01 to 0.82; semester six, aPR 1.33, 95% CI 1.06 to 1.67; semester seven, aPR 1.64, 95% CI 1.16 to 2.30), as was the lower prevalence among participants who spent most of their time in theoretical and practical teaching events (aPR 0.10, 95% CI 0.02 to 0.42). The associations with age (aPR 0.93 per year, 95% CI 0.84 to 1.03), placement outside a university hospital (aPR 0.70, 95% CI 0.42 to 1.16) and no placement in self-employed midwifery practice (aPR 0.58, 95% CI 0.29 to 1.16) were not observed under the stricter criterion, whereas no placement in a private hospital was associated with a higher prevalence (aPR 2.20, 95% CI 1.31 to 3.69), an estimate based on only 15 participants without such a placement. Among participants who did not give uniform barrier ratings, 32.0% met the stricter criterion (Table 7).

**Table 7:** Self-reported ability in EBP activities under the main definition (at least three of four items) and the stricter post hoc definition (all four items)

	At least 3 of 4 items (main definition)	All 4 items (post hoc sensitivity analysis)
Prevalence, *n*/*N* (%, 95% CI)	149/272 (54.8, 48.8 to 60.6)	128/270 (47.4, 41.5 to 53.4)
Prevalence excluding uniform responders, %	42.2	32.0
Adjusted prevalence ratios (95% CI)		
Semester 5 (reference)	1.00	1.00
Semesters 2 to 4	0.35 (0.20 to 0.62)	0.09 (0.01 to 0.82)
Semester 6	1.18 (0.98 to 1.43)	1.33 (1.06 to 1.67)
Semester 7	0.98 (0.79 to 1.22)	1.64 (1.16 to 2.30)
Age, per year	1.12 (1.06 to 1.18)	0.93 (0.84 to 1.03)
Mainly theoretical and practical teaching events	0.43 (0.28 to 0.66)	0.10 (0.02 to 0.42)
No placement in self-employed midwifery practice	0.42 (0.25 to 0.71)	0.58 (0.29 to 1.16)
No placement in a university hospital	1.91 (1.40 to 2.62)	0.70 (0.42 to 1.16)
No placement in a private hospital	1.08 (0.71 to 1.66)	2.20 (1.31 to 3.69)
No placement in a midwife-led birth centre	1.03 (0.77 to 1.40)	1.00 (0.68 to 1.49)

Items were counted as endorsed for “agree” or “strongly agree” (“applies” or “fully applies” for the item on critically evaluating a research article); missing responses were never counted as endorsement, and participants were classified only when the available responses determined the result. Under the stricter criterion, no participant in semesters 2 or 3 and 1 participant in semester 4 met the outcome, so semesters 2 to 4 were combined in both models to allow comparison; estimates for the main definition therefore differ slightly from those of the model with separate semesters. Estimates were obtained using modified Poisson regression with robust variance, adjusted for all variables shown; the participant in semester 8 was excluded from the models. Uniform responders gave the same rating to all 10 barrier items (*n* = 99)

*CI* confidence interval

## Discussion

This study examined several aspects of evidence-based practice (EBP) among undergraduate midwifery students in Germany after the transition to fully academic midwifery education. Of the 271 students who provided valid responses to the knowledge item, 64.9% correctly identified EBP as the integration of scientific evidence, practical expertise, and client preferences. Attitudes were predominantly positive across all nine attitude items. However, understanding and valuing EBP did not result in confidence in the specific competencies required for its application, nor in the consistent use of these competencies during clinical placements.

Direct comparisons with pre-registration midwifery students are limited because research on EBP in midwifery is less developed than in nursing or other health professions. The most comparable German studies include physiotherapists, nurses, and midwives collectively, and similarly report positive attitudes toward EBP alongside challenges in translating evidence into practice [40]. Reviews involving pre-registration midwifery students indicate that EBP instruction enhances knowledge and attitudes, yet students perceive a disconnect between their education and clinical observations [3, 16, 17], consistent with the barriers identified in this study. Research in nursing and other health student groups demonstrates that positive attitudes toward EBP do not reliably correspond to strong appraisal or implementation competencies [13, 41–43]. Such comparisons should be interpreted cautiously, given differences in professional roles, curriculum content, clinical exposure, and EBP instruction across professions. This caution is particularly relevant in Germany, where academic midwifery education commenced only in 2020 [7, 8]. The finding that approximately two-thirds of students recognised the comprehensive definition of EBP suggests that most understood the integrated concept, which is necessary for practice but does not confirm their ability to implement it. However, because the knowledge assessment relied on a single recognition item, it does not determine whether students can complete the full EBP process. Students’ confidence in understanding the EBP model and describing its steps also differed markedly between semesters 2 to 4 and semesters 5 to 7, but these items measure self-reported confidence rather than objectively tested knowledge.

The attitude items provide additional insight into students’ understanding of EBP. While 93.0% agreed that EBP improves patient outcomes and 90.1% affirmed that research is useful in daily practice, fewer recognised that EBP incorporates patient preferences (68.0%) or clinical experience (70.4%). This pattern indicates that some students perceive EBP primarily as the application of research evidence, rather than as the integration of research, professional expertise, and patient values. This distinction is significant in midwifery, where decisions require balancing evidence, individualised care, and informed choice. Therefore, instruction should address not only how to locate and appraise research, but also how to integrate all three EBP components in practice.

Another notable finding is the discrepancy between general confidence in EBP and confidence in specific research competencies. Approximately half to three-fifths of participants reported the ability to apply search strategies in scientific databases (58.5%), critically appraise literature (55.2%), identify appropriate study designs (52.6%), or critically evaluate research articles (51.8%). Students reported confidence in searching for scientific evidence and conducting scientific work at 45.6% and 48.7%, respectively. These figures represent students’ self-assessed abilities rather than objectively measured competence, as no performance-based assessments were conducted. Self-reported confidence may either overestimate or underestimate actual skills. Nonetheless, these results indicate that recognising the definition of EBP did not translate into confidence in executing EBP-related tasks. Competencies such as critical appraisal and applying research methods are advanced skills that typically require repeated practice and feedback, rather than classroom instruction alone [13, 43–46].

The contrast is even more pronounced in reported behaviours. While 52.9% agreed that research findings should frequently be utilised during clinical placements, only 12.2% always or frequently critically appraised the literature they used, and just 8.5% always or frequently applied research findings in practice. These figures do not reflect independent professional performance, as participants remained in supervised clinical education. The results indicate that valuing research use is considerably more prevalent among students than the actual appraisal and application of evidence in clinical decision-making, consistent with previous studies that report a gap between valuing EBP and consistently implementing all of its components [13, 43–46].

The adjusted analysis illustrates how outcomes differed between semesters. Compared to semester five, good EBP knowledge was significantly less prevalent in semester two (aPR 0.05, 95% CI 0.01 to 0.37) and semester three (aPR 0.17, 95% CI 0.06 to 0.44), and moderately less prevalent in semester four (aPR 0.70, 95% CI 0.49 to 0.99). Prevalence was higher in semester six (aPR 1.21, 95% CI 1.01 to 1.46), and the estimate for semester seven was similar, though with a wider confidence interval (aPR 1.23, 95% CI 0.97 to 1.56). Self-reported competencies exhibited a similar gradient in the crude analysis, although after adjustment only semester four differed clearly from semester five; when all four abilities were required, the gradient was more pronounced (Table 7). This gradient is consistent with a curriculum that progressively develops EBP, but the study design cannot distinguish educational progression from other potential explanations. Students in different semesters represent different cohorts who entered programs at various stages of development; semester distribution varied across universities, potentially reflecting institutional differences in the estimates; and research methods are introduced at different points in the curriculum [8], so the marked increase between semesters 3 and 5 may reflect the timing of specific modules rather than gradual development. Including university as a variable did not substantially alter the semester estimates, suggesting that institutional differences are not the sole explanation, although institutional effects cannot be excluded with only seven sites. The observed gradient also cannot be interpreted as individual change, as the analysis compared different students at a single time point. Longitudinal studies are necessary to determine how EBP competencies develop within individual students.

Learning context was less strongly associated with the primary outcome than semester of study. In the crude analysis, the main area of learning or work and certain placement experiences appeared to be strongly associated with EBP knowledge, but these associations diminished substantially after adjustment. Students who spent most of their time in theoretical and practical teaching events did not differ significantly from those primarily engaged in mother and child health care (aPR 0.72, 95% CI 0.50 to 1.04), and the initial associations with placements in midwife-led birth centres and self-employed midwifery practice did not persist. Thus, much of the observed difference between settings was attributable to differences in semester of study. In this sample, recognition of the EBP definition was not dependent on the primary setting of practical education. However, interpret this observation with caution, as the variable captured where students spent most of their learning time rather than exposure to specific educational interventions, and the study could not assess what occurred within individual placements.

Secondary analyses suggest that learning context may influence how students assess their own EBP abilities. Students who spent most of their time in theoretical and practical teaching events reported lower competence and less favourable attitudes after adjustment, and the association with competence persisted when all four abilities were required (Table 7). Students without placement experience in self-employed midwifery practice also reported lower competence and less favourable attitudes, although this association was not observed for competence under the stricter definition. One possible explanation is that students focused on theory may become more aware of the complexity of EBP and thus judge their competencies more critically, whereas clinical experience may enhance confidence through practical application. Conversely, students without a university hospital placement reported higher competence, which contrasts with the expectation that academic centres provide greater research exposure. Several factors may contribute to this finding. University hospitals are large and organizationally complex, with high caseloads, multiple professional groups, standardised protocols, and frequently changing supervisors, which may limit opportunities for students to assume responsibility or discuss care rationales. In smaller hospitals and midwife-led settings, students are often supervised by the same midwife over extended periods and may receive greater autonomy, which can foster confidence. Students in university hospitals may also encounter research-active staff and higher expectations regarding EBP, leading them to evaluate their abilities more critically. Because only 26 participants were not placed in a university hospital, the estimate is imprecise, and the current data cannot test these explanations; further research should examine supervision structures and student autonomy. Moreover, this association was not observed in the post hoc sensitivity analysis requiring endorsement of all four practical abilities (Table 7); together with the changes for age and for placement in self-employed midwifery practice, this indicates that the covariate associations for the competence outcome depend on the chosen threshold and should be regarded as tentative. These patterns may also reflect differences between student groups, course timing, or unmeasured variables. Because attitude and competence scores came from an adapted questionnaire rather than validated German midwifery scales and relied on self-report, these findings should be considered secondary and interpreted with caution.

The barriers students identified help explain why favourable attitudes and moderate knowledge levels do not consistently lead to evidence use. The most frequently cited barrier was insufficient English-language skills (87.4%), followed by lack of collegial support in clinical settings (86.2%) and difficulty transferring evidence into practice (85.4%). Insufficient time (70.5%) and lack of institutional or technical resources (69.8%) were also commonly rated as highly relevant. These findings indicate that students perceive barriers at multiple interconnected levels, including individual competencies and language, translating knowledge into practice, clinical supervision, and the broader institutional learning environment.

However, interpreting barrier ratings requires caution. Even the lowest-rated items received scores of 8 to 10 from approximately two-thirds of participants, and every item had a median rating of 9 or 10. This pattern may indicate that barriers are genuinely widespread, but it also suggests a ceiling effect: a 1 to 10 relevance scale labelled only at its endpoints may encourage high ratings and limit discrimination between challenges. Two observations support this interpretation. Lack of interest was rated 8 to 10 by 67.4% of participants, which is difficult to reconcile with the consistently favourable attitudes reported in Table 4, and the statement that introducing EBP is not feasible was rated nearly as highly as external barriers. Some participants may have rated the importance of each topic rather than its relevance as a personal barrier, and 36.4% assigned the same rating to all 10 items, almost always the maximum. Such uniform responses may reflect low effort, and identical records could include repeated submissions, which cannot be excluded in an anonymous survey. Uniform responders were also more likely to meet every outcome definition, and excluding them reduced all prevalence estimates by 13 to 20% points, while maintaining the semester gradient and the ranking of the three leading barriers. The means in Table 6 allow for ranking of items, but the absolute percentages should not be interpreted as prevalence estimates. Future studies should employ fully labelled response categories, ranking tasks, or forced-choice formats to better distinguish between barriers.

English-language difficulties are particularly significant within the German education system. Because most international health research is published in English, students may grasp EBP principles but still struggle to access, comprehend, and appraise original research. This issue is infrequently addressed in the EBP literature, which is itself predominantly in English. Many EBP competency frameworks assume that learners can locate and evaluate international research, often overlooking the language demands imposed [6, 47]. Consequently, EBP education in countries where English is not the primary language of instruction may need to incorporate academic English and scientific literacy [48].

The finding that 85.4% of students reported difficulty implementing evidence in practice aligns with international research showing that knowledge translation is a distinct EBP competency, not an automatic outcome of research training [49]. Students may learn to formulate questions, search databases, and appraise research in the classroom, yet face challenges applying these skills in clinical settings where routines, local protocols, or supervisor expectations differ from the evidence. This underscores the need to teach EBP not only as theoretical knowledge, but also through opportunities to apply evidence-based reasoning in authentic clinical situations.

The lack of support from colleagues in clinical settings is particularly relevant in the German dual model of midwifery education, where much of the training occurs in supervised clinical environments. Previous studies have also identified mentorship, role models, and supervisor engagement as critical factors in translating EBP education into clinical learning [43, 50, 51]. When students are exposed to evidence-based reasoning at university but do not see supervisors using or discussing research in practice, the link between academic EBP instruction and clinical care becomes harder to establish. Although this study cannot detail the support available at individual placements, the frequency with which students identified this barrier suggests that the relationship between university teaching and clinical supervision requires further investigation.

Insufficient time and limited resources further show that EBP implementation does not depend solely on individual student competence. Students need access to databases, full-text articles, dedicated learning time, functional digital tools, and opportunities to discuss evidence with instructors and supervisors [15]. However, interpret these barriers cautiously. Several items were adapted from instruments designed for qualified professionals, and students in supervised placements may perceive time, resources, and support differently than independent practitioners. Consequently, the results reflect students’ perceptions of barriers within their learning environments rather than providing an objective assessment of institutional support for EBP.

Collectively, these findings characterise students who value EBP and recognise its definition, report moderate self-assessed competencies, and infrequently engage in regular appraisal and application of evidence due to substantial barriers. Given the cross-sectional design and the ongoing evolution of German midwifery education, these results are best interpreted as a snapshot of a system in transition, rather than as an evaluation of the reform’s effectiveness.

### Recommendations

The recommendations from this study are designed to guide curriculum development, not to function as evaluated interventions. These recommendations are especially pertinent to midwifery education, where students must integrate research evidence, clinical expertise, and individual preferences when deciding on care, intervention, and referral.

First, although attitudes towards evidence-based practice (EBP) were highly positive, self-reported competencies were only moderate. Curricula should therefore emphasise repeated, supervised practice in literature searching, critical appraisal of studies, recognition of study designs, and applying evidence to clinical situations, supported by structured feedback [52–54]. Familiarity with EBP concepts alone is insufficient to develop confidence in the complex competencies required for evidence-based decision-making.

Second, recognition of the EBP definition was considerably lower in semesters two and three compared to later semesters. Regardless of the underlying cause, the low recognition rates in early semesters support integrating EBP as a continuous element throughout the programme and reinforcing it across both theoretical and clinical phases, rather than limiting it to isolated research or EBP modules [52, 55]. Due to the cross-sectional design, this pattern cannot be interpreted as progression within individual students.

Third, lack of support from colleagues at placement sites emerged as one of the most significant barriers. Bridging the gap between university instruction and clinical practice is essential for the credibility of EBP teaching. Stronger partnerships between universities and placement sites, better preparation of clinical supervisors in EBP, and structured evidence-based activities during placements would give students opportunities to apply evidence in real clinical situations [50, 51]. This is particularly important in the German dual system, where theoretical and clinical learning occur concurrently, and a disconnect between theory and practice would undermine both.

Fourth, limited proficiency in scientific English was the most significant barrier, suggesting that academic language support should be integrated into EBP education rather than provided separately. Subject-specific support in reading and interpreting scientific English, along with access to high-quality German-language summaries or appraised evidence where available, may help reduce linguistic barriers to engaging with international research literature.

Fifth, the transition to university-based midwifery education remains ongoing and would benefit from continuous evaluation. Systematic monitoring of EBP teaching, sequencing, and assessment across the programme; regular exchange between institutions on curriculum design and placement organisation; and structured collaboration between academic and clinical components would help identify and share effective approaches as the system evolves.

Finally, many students reported insufficient time and resources, indicating that developing individual competence alone is inadequate. Universities and clinical placement sites should ensure that students have access to scientific databases, full-text articles, and digital tools, as well as dedicated time to engage with research evidence during both academic and clinical phases of the programme.

### Strengths and limitations of the study

To the best of the authors’ knowledge, this is the first multi-site study to examine EBP knowledge, attitudes, self-reported competencies, behaviours, and barriers among undergraduate midwifery students within Germany’s new academic education system. The study provides baseline data on a system that is still evolving following academisation. These observations provide a reference point for future studies to monitor the academisation of midwifery programmes in Germany and its effect on students’ EBP competencies. The sample included 272 eligible students from seven universities across five federal states, representing both public and private institutions. The questionnaire was adapted from established EBP instruments, facilitating careful comparison with international research.

Several limitations should be considered. First, as the study is cross-sectional, it cannot support conclusions regarding causation or changes within individuals. The observed differences between semesters and learning settings reflect differences between groups at a single point in time, rather than changes within the same students.

Second, participation was voluntary and organised through the universities. Only 7 of the 48 universities offering midwifery programmes in Germany participated; the response rate could not be calculated, and two institutions accounted for more than half of the sample. Students with a particular interest in research or EBP are more likely to respond to a voluntary survey on the subject, and universities that agreed to participate may have more developed EBP teaching. Both factors likely inflate estimates of knowledge, self-reported competence, and positive attitudes; thus, prevalence figures are more likely to overestimate than underestimate the values for the participating programmes and should not be read as national estimates. The participating universities included both public and private institutions, as well as universities of applied sciences and medical faculties. However, because participation depended on institutional agreement, these universities may differ from non-participating programmes in unmeasured ways, including the extent of EBP teaching development. The eligibility criteria, which required completion of an EBP or research methods module and a clinical placement, excluded students with the least exposure and would have a similar effect. For barrier ratings, the direction of bias is less clear, as students engaged with EBP may also be more aware of obstacles. Associations between variables are generally less affected by selection than prevalence estimates, but they may not apply to all midwifery students in Germany.

Third, most data on attitudes, competencies, behaviours, and barriers were self-reported, making them susceptible to social desirability bias and variations in self-perception. Students with greater EBP knowledge may assess their competencies more critically, while others may overestimate their abilities. Consequently, these measures reflect confidence rather than objectively assessed competence, and the two may differ substantially.

Fourth, the primary knowledge outcome was based on a single multiple-choice item requiring recognition of the comprehensive definition of EBP. This approach captures conceptual knowledge rather than the ability to formulate questions, search for evidence, appraise research, or apply evidence in clinical decisions, and the binary classification oversimplifies a complex construct. Additionally, because participants completed the survey online and without supervision, the authors could not verify whether they answered the knowledge items independently.

Fifth, the questionnaire was adapted from instruments originally developed for nursing students or qualified health professionals. The authors reworded items, removed three, added four, and translated the instrument without formal cross-cultural validation [28]. These modifications may have altered the measurement properties of the original scales, and no psychometric evaluation beyond internal consistency was conducted. The adapted instrument should therefore be considered unvalidated in this population; its scores cannot be directly compared with published EBPQ or S-EBPQ values, and the investigators determined the cut-offs for binary outcomes rather than deriving them from validation studies. For the competence outcome in particular, the threshold has not been validated against observed EBP performance, and dichotomising the responses loses information; the proportion meeting the criterion should therefore not be read as the proportion of students with demonstrated competence, and several covariate associations changed when the stricter threshold was applied. The items may also fail to capture all EBP aspects specific to undergraduate midwifery education. Students in supervised placements may interpret items on barriers such as workplace conditions and collegial support differently from qualified professionals.

Sixth, interpret the summed scores for attitudes and self-reported competencies with caution. Internal consistency was 0.97 for the attitude scale, which may indicate redundant items, and 0.96 and 0.95 for the competence and barrier scales, respectively. However, as the questionnaire was not originally developed for German midwifery students, these findings are exploratory rather than definitive. Additionally, barrier ratings were clustered at the top of the scale, limiting discrimination between items, and approximately one-third of participants provided uniform ratings; excluding these responses lowered all prevalence estimates.

Finally, the study included only undergraduate students, so it cannot determine whether these patterns persist after graduation or how effectively graduates apply EBP in professional practice. Longitudinal research following students beyond graduation is necessary to address this limitation.

### Conclusions

Among 272 undergraduate midwifery students at seven German universities, approximately two-thirds identified the comprehensive definition of evidence-based practice, and attitudes towards EBP were consistently favourable. However, self-reported competencies were moderate, and regular appraisal and application of research findings during clinical placements remained uncommon. The semester of study was more strongly associated with recognition of the EBP definition than any specific learning or placement setting. Cohort and institutional differences may also contribute to this pattern. Most students reported substantial barriers, particularly limited proficiency in scientific English, lack of support at placement sites, difficulty transferring evidence into practice, insufficient time, and limited resources.

As the study is cross-sectional, utilised a self-selected sample, relied primarily on self-report using an adapted instrument not validated in this population, and measured knowledge with a single objective item, the findings should be interpreted as baseline data for the participating students rather than as a measure of clinical competence or the success of the national education reform. These results provide a reference point for assessing the ongoing development of EBP teaching as university-based midwifery education in Germany evolves.

### Implications for future research

Future research should track students from the beginning of the midwifery programme through graduation and into early professional practice. This approach would help determine whether the differences observed between semesters in this study reflect genuine growth in EBP knowledge and competencies over time or differences between cohorts and programme structures.

Further research is needed to evaluate specific approaches to teaching EBP, particularly studies that combine self-reported outcomes with objective assessments of literature searching, critical appraisal, evidence interpretation, and application of evidence to clinical decisions [52]. Controlled studies could determine whether teaching EBP throughout the programme using clinical activities, simulation, case-based learning, or structured supervision is more effective than conventional classroom teaching.

Further research should examine the interaction between university teaching and clinical learning environments, particularly how clinical supervisors’ EBP competencies, access to evidence resources, and opportunities for evidence-based discussion during placements influence students’ development.

The language barrier identified in this study warrants further investigation. Future studies could assess students’ comprehension of scientific English and its impact on their ability to search for, appraise, and apply evidence in settings where English is not the language of instruction. Additionally, research could evaluate whether targeted language support increases engagement with EBP.

Finally, future studies should include newly qualified and experienced midwives to determine whether EBP competencies acquired during undergraduate education are retained and utilised after graduation, and to identify workplace factors that support or hinder the use of EBP in professional practice.

## Supplementary Information

Supplementary Material 1.

## Data Availability

The datasets used and analyzed during the current study are available from the corresponding author on reasonable request, subject to ethical and data protection considerations.

## Abbreviations

aPR: Adjusted prevalence ratio
APC: Article Processing Charge
BDSG: Bundesdatenschutzgesetz (German Federal Data Protection Act)
CI: Confidence interval
cPR: Crude prevalence ratio
CVI: Content Validity Index
EBP: Evidence-based practice
EBPQ: Evidence-Based Practice Questionnaire
GDPR: General Data Protection Regulation
HebStPrV: Studien- und Prüfungsverordnung für Hebammen (Study and Examination Regulation for Midwives)
IQR: Interquartile range
REDCap: Research Electronic Data Capture
SD: Standard deviation
S-EBPQ: Student Evidence-Based Practice Questionnaire
STROBE: Strengthening the Reporting of Observational Studies in Epidemiology
