# Supplementary material for: Knowledge, attitudes, self-reported competencies and perceived barriers regarding evidence-based practice among undergraduate midwifery students in Germany: a cross-sectional study
Source: BMC Med Educ. 2026 Nov 13;26:1861. doi: 10.1186/s12909-026-10846-y (PMC13769167; doi:10.1186/s12909-026-10846-y)
Supplement: Supplementary file 1 [file 12909_2026_10846_MOESM1_ESM.pdf]

Additional file 1. Extent of missing data in the analysis sample (N = 272)

Variable	Missing, n	Valid n	% missing
Institution	15	257	5.5
Pre-university qualification	10	262	3.7
Barrier: lack of institutional resources	7	265	2.6
Barrier: competence in computers and technology	7	265	2.6
Barrier: systematic literature searching	5	267	1.8
Barrier: introducing EBP not feasible	5	267	1.8
Area in which most work was carried out	4	268	1.5
Competence item: critically appraise literature	4	268	1.5
Barrier: insufficient time	4	268	1.5
Barrier: critically evaluating literature	4	268	1.5
Barrier: transfer of findings into practice	4	268	1.5
Barrier: no support from colleagues	3	269	1.1
Barrier: limited English-language skills	2	270	0.7
Barrier: lack of interest	2	270	0.7
Attitude: EBP takes account of clinical experience	2	270	0.7
Competence item: confidence in searching for evidence	2	270	0.7
Belief: EBP improves outcomes for mothers and children	2	270	0.7
Behaviour: critically appraises literature used	2	270	0.7
Attitude: use of EBP is necessary	1	271	0.4
Competence item: confidence in conducting scientific work	1	271	0.4
Gender	1	271	0.4
EBP definition item (primary outcome)	1	271	0.4

Variables with no missing responses are not listed. Administrative fields, including the survey identifier and the closing acknowledgement, are excluded. No variable had more than 15 of 272 missing responses and no outcome item had more than 7.

Additional file 2. Adaptation of the EBPQ and S-EBPQ for German undergraduate midwifery students

This file documents how the survey instrument was derived from the Evidence-Based Practice Questionnaire (EBPQ; Upton and Upton, 2006) and the Student Evidence-Based Practice Questionnaire (S-EBPQ; Upton et al., 2016), together with the items created specifically for the German B.Sc. Midwifery Science context.

Three adaptation decisions were made. Practice-behaviour items were reworded from a qualified-practitioner frame to the perspective of a student on clinical placement. Three items concerning prescribing or independent clinical decision-making were removed. Four barrier items relevant to German higher education were added following the qualitative sub-study. The original item numbers were not carried forward into the final instrument, and the four newly added barrier statements are not separately identifiable among the ten barriers in the final questionnaire; item-level origins are therefore reported only where they can be established from the study record.

Table S2.1. Summary of instrument adaptation

Domain	Source	Status	What was done
EBP practice behaviour	EBPQ / S-EBPQ	Changed	Practice items retained conceptually but reworded from qualified-practitioner language to the perspective of a midwifery student on clinical placement.
Attitudes towards EBP	EBPQ / S-EBPQ	Retained and adapted	Core attitude constructs retained; wording contextualised to midwifery education and midwifery practice.
EBP knowledge and skills	EBPQ / S-EBPQ plus study adaptation	Retained and adapted	Core knowledge and skills constructs retained and supplemented with objective and contextual questions suitable for undergraduate students.
Prescribing and autonomous clinical decision-making	Original source instruments	Removed	Three items removed as not applicable to the role of German undergraduate midwifery students. Original item numbers and wording are not recorded in the study documentation.
Perceived barriers	Existing barrier content plus study-specific additions	Changed and new	Ten barrier statements were used in the final questionnaire. Four were added for the German higher-education context following the qualitative sub-study; the

Domain	Source	Status	What was done
			four are not individually identified in the study documentation.
Sociodemographic and educational characteristics	Study-specific	Newly created	Items on gender, age, university, pre-university qualification, semester of study, clinical placement and learning or work context were created for the present study.

Table S2.2. Final questionnaire item inventory and adaptation status

ID	Section	Final item or construct	Source	Status
1.1	Sociodemographic	Gender	Study-specific	Newly created
1.2	Sociodemographic	Age	Study-specific	Newly created
1.3	Sociodemographic	University attended	Study-specific	Newly created
1.4	Sociodemographic	Place where the university entrance qualification was obtained	Study-specific	Newly created
1.5	Educational	Semester of study	Study-specific	Newly created
1.6	Educational and clinical	Main clinical placement setting	Study-specific	Newly created
1.7	Educational and work context	Area in which most time was spent	Study-specific	Newly created
2.1	EBP knowledge	Definition of EBP (primary outcome)	Adapted instrument	Retained and adapted
2.2	EBP knowledge	Knowledge of the EBP model	Adapted instrument	Retained and adapted
2.3	EBP knowledge	Identification of the EBP process steps	Adapted instrument	Retained and adapted
2.4	EBP knowledge	Best study design for evaluating calcium supplementation to prevent pre-eclampsia	Study-adapted objective item	New and adapted
3.1	Behaviour	Frequency of using research findings during clinical placements	EBPQ / S-EBPQ practice construct	Changed
3.2	Behaviour	Frequency of critically appraising the literature used	EBPQ / S-EBPQ practice construct	Changed
3.3	Behaviour	Frequency of applying research findings in practice	EBPQ / S-EBPQ practice construct	Changed
3.4	Competence	Ability to apply search strategies in scientific databases	EBPQ / S-EBPQ skills construct	Retained and adapted
3.5	Competence	Ability to appraise literature for quality and significance	EBPQ / S-EBPQ skills construct	Retained and adapted
3.6	Competence	Ability to identify the appropriate study design	EBPQ / S-EBPQ skills construct	Retained and adapted

ID	Section	Final item or construct	Source	Status
3.7	Competence	Ability to critically evaluate a research article	EBPQ / S-EBPQ skills construct	Retained and adapted
3.8	Confidence	Confidence in searching for scientific evidence	EBPQ / S-EBPQ skills construct	Retained and adapted
3.9	Confidence	Confidence in conducting scientific work	EBPQ / S-EBPQ skills construct	Retained and adapted
3.10	Belief	EBP improves outcomes for mothers and children	EBPQ / S-EBPQ construct	Retained and adapted
4.1	Attitude	I need to strengthen my use of EBP in daily practice	EBPQ / S-EBPQ attitude construct	Retained and adapted
4.2	Attitude	EBP improves outcomes for patients	EBPQ / S-EBPQ attitude construct	Retained and adapted
4.3	Attitude	Literature and research are useful for EBP in daily practice	EBPQ / S-EBPQ construct	Retained and adapted
4.4	Attitude	EBP should be a core component of midwifery studies	S-EBPQ student context	Retained and adapted
4.5	Attitude	The use of EBP is necessary in midwifery practice	EBPQ / S-EBPQ attitude construct	Retained and adapted
4.6	Attitude	Stronger EBP competence improves my work as a midwife	Adapted instrument	Retained and adapted
4.7	Attitude	EBP is an important part of midwifery practice	EBPQ / S-EBPQ attitude construct	Retained and adapted
4.8	Attitude	EBP takes account of clinical experience	EBPQ / S-EBPQ construct	Retained and adapted
4.9	Attitude	EBP takes account of patient preferences and values	EBPQ / S-EBPQ construct	Retained and adapted
5.1	Barriers	Whether barriers to using EBP in clinical practice exist	Study-adapted barrier section	Retained and adapted
5.2a	Barrier	Introducing EBP is not feasible in midwifery practice	Existing and study-adapted barrier pool	Provenance not documented
5.2b	Barrier	Insufficient time	Existing and study-adapted barrier pool	Provenance not documented
5.2c	Barrier	Lack of institutional resources (computers, databases)	Existing and study-adapted barrier pool	Provenance not documented
5.2d	Barrier	Limited competence in using computers and technology	Existing and study-adapted barrier pool	Provenance not documented

ID	Section	Final item or construct	Source	Status
5.2e	Barrier	Limited competence in systematic literature searching	Existing and study-adapted barrier pool	Provenance not documented
5.2f	Barrier	Limited competence in critically evaluating literature	Existing and study-adapted barrier pool	Provenance not documented
5.2g	Barrier	Difficulty transferring literature findings into practice	Existing and study-adapted barrier pool	Provenance not documented
5.2h	Barrier	Limited English-language skills	Existing and study-adapted barrier pool	Provenance not documented
5.2i	Barrier	No support from colleagues at the placement site	Existing and study-adapted barrier pool	Provenance not documented
5.2j	Barrier	Lack of interest	Existing and study-adapted barrier pool	Provenance not documented

The final questionnaire comprised 41 items: 7 sociodemographic and educational, 4 objective knowledge, 10 self-reported competencies and behaviours, 9 attitudes and 11 barrier items (one screening question and ten rated barriers). Section 5 barriers are listed in the order used in the questionnaire; Table 6 of the main manuscript orders the same items by the proportion rating them 8 to 10.

Table S2.3. Items removed from the source instruments

Items	Original content area	Status	Reason
R1 to R3	Three items concerning prescribing or autonomous clinical decision-making	Removed	Not applicable to the role and scope of undergraduate midwifery students in Germany. The original item numbers and exact wording are not recorded in the study documentation.

Additional file 3. Comparison of participating and non-participating B.Sc. Midwifery Science programmes

This file describes the institutional characteristics of the seven programmes that participated in the quantitative survey and sets them against the wider German programme landscape. Participating institutions are reported in anonymised form to preserve the confidentiality of the study sites.

Table S3.1. Aggregate comparison of participating and non-participating programmes

Characteristic	Participating programmes	Non-participating and national programmes
Number of institutions	7	41 of the 48 accredited primary-qualifying B.Sc. midwifery programmes identified in the sampling frame
Federal-state coverage	5 federal states: Bavaria, Rhineland-Palatinate, Berlin, Lower Saxony and North Rhine-Westphalia	National provision across all 16 federal states
Public or private status	5 public and 2 private	Both public and private providers nationally
Year of programme establishment	Winter semester 2019/20 (2 programmes), 2020/21 (1) and 2021/22 (4)	Includes pre-reform model programmes and programmes established after the 2020 reform
Accreditation and programme type	Accredited primary-qualifying B.Sc. midwifery programmes	Accredited primary-qualifying B.Sc. midwifery programmes
Study model	Dual, practice-integrated B.Sc. midwifery	Same statutory programme context nationally

Table S3.2. Characteristics of the seven participating programmes

Code	Federal state	Status	Programme established
University A	Bavaria	Public	Winter semester 2019/20
University B	Rhineland-Palatinate	Public	Winter semester 2021/22
University C	Berlin	Public	Winter semester 2021/22
University D	Lower Saxony	Public	Winter semester 2020/21
University E	Lower Saxony	Public	Winter semester 2021/22
University F	North Rhine-Westphalia	Private	Winter semester 2021/22
University G	Bavaria	Private	Winter semester 2019/20

All seven programmes are accredited primary-qualifying B.Sc. midwifery programmes. Institution names are omitted to preserve the anonymity of the participating study sites. Public or private status and year of establishment were taken from official institutional and state sources.

Table S3.3. Federal-state coverage of the seven participating programmes

Federal state	Participating programmes	Comment
Bavaria	2	One public and one private programme
Rhineland-Palatinate	1	Public programme
Berlin	1	Public programme
Lower Saxony	2	Both public programmes
North Rhine-Westphalia	1	Private programme
Remaining 11 federal states	0	No participating programme

Three respondents in the main analysis recorded a public university in North Rhine-Westphalia that was not among the seven participating study sites. These responses were retained in the analysis sample and are footnoted in Table 1 of the main manuscript.

Additional file 4. Factors associated with the secondary outcomes

Table S4.1. Crude and adjusted prevalence ratios for good self-reported EBP competence and for a positive attitude towards EBP

Variable	cPR (95% CI)	p	aPR (95% CI)	p
Good self-reported EBP competence (adjusted n = 267)				
Age, per year	1.20 (1.13 to 1.27)	<0.001	1.12 (1.06 to 1.19)	<0.001
Semester 2 (vs 5)	0.12 (0.03 to 0.47)	0.002	0.61 (0.18 to 2.09)	0.433
Semester 3 (vs 5)	0.13 (0.04 to 0.40)	<0.001	0.53 (0.18 to 1.52)	0.236
Semester 4 (vs 5)	0.12 (0.05 to 0.32)	<0.001	0.27 (0.13 to 0.55)	<0.001
Semester 6 (vs 5)	1.50 (1.25 to 1.79)	<0.001	1.17 (0.96 to 1.41)	0.114
Semester 7 (vs 5)	1.52 (1.28 to 1.82)	<0.001	0.96 (0.77 to 1.21)	0.738
Teaching events (vs mother and child health care)	0.11 (0.06 to 0.18)	<0.001	0.41 (0.26 to 0.66)	<0.001
No placement in self-employed practice (vs yes)	0.11 (0.07 to 0.19)	<0.001	0.39 (0.22 to 0.71)	0.002
No placement in a university hospital (vs yes)	1.47 (1.15 to 1.87)	0.002	1.95 (1.39 to 2.74)	<0.001
No placement in a private hospital (vs yes)	0.97 (0.60 to 1.58)	0.909	1.08 (0.66 to 1.78)	0.757
No placement in a birth centre (vs yes)	0.21 (0.15 to 0.29)	<0.001	1.02 (0.75 to 1.39)	0.908
Positive attitude towards EBP (adjusted n = 264)				
Age, per year	1.18 (1.11 to 1.24)	<0.001	1.08 (1.03 to 1.14)	0.002
Semester 2 (vs 5)	Not estimable a		Not estimable a	
Semester 3 (vs 5)	0.21 (0.09 to 0.48)	<0.001	0.38 (0.14 to 0.99)	0.047
Semester 4 (vs 5)	0.40 (0.24 to 0.65)	<0.001	0.67 (0.43 to 1.06)	0.088
Semester 6 (vs 5)	1.43 (1.22 to 1.68)	<0.001	1.11 (0.99 to 1.25)	0.073
Semester 7 (vs 5)	1.40 (1.18 to 1.66)	<0.001	0.98 (0.83 to 1.18)	0.865
Teaching events (vs mother and child health care)	0.19 (0.13 to 0.28)	<0.001	0.62 (0.39 to 0.99)	0.047
No placement in self-employed practice (vs yes)	0.20 (0.14 to 0.29)	<0.001	0.59 (0.37 to 0.94)	0.027
No placement in a university hospital (vs yes)	0.83 (0.56 to 1.23)	0.348	0.84 (0.58 to 1.21)	0.345

Variable	cPR (95% CI)	p	aPR (95% CI)	p
No placement in a private hospital (vs yes)	1.13 (0.78 to 1.64)	0.523	1.29 (0.87 to 1.91)	0.204
No placement in a birth centre (vs yes)	0.28 (0.21 to 0.37)	<0.001	0.99 (0.77 to 1.27)	0.941

cPR, crude prevalence ratio; aPR, adjusted prevalence ratio; CI, confidence interval. Estimated by modified Poisson regression with robust variance, using the same specification as the primary model reported in Table 3 of the main manuscript. a No participant in semester two met the attitude outcome, so the estimate is not identified.
